# Supplementary material for: The genome sequence of the Regal Piercer, Pammene regiana (Zeller, 1849) (Lepidoptera: Tortricidae)
Source: Wellcome Open Res. 2026 Apr 21;11:232. [Version 1] doi: 10.12688/wellcomeopenres.26362.1 (PMC13270186; doi:10.12688/wellcomeopenres.26362.1)
Supplement: Peer review history

# Reviewer report: Reviewer response for version 1

**DOI:** 10.21956/wellcomeopenres.29033.r157326

**Published:** 2026-06-22

**Authors:** Christophe Klopp

**Affiliations:** 1 INRAE, Castanet-Tolosan, France

The authors present the first chromosomal assembly of the Pammene regiana genome. The methods are usual and the article is standard. 

 The authors should indicate if hifiasm was run with Hi-C or not.

 The authors should have provided hap1 and hap2 which are easier to exploit in further analyses than primary and alternate which are now outdated. 

 The authors should give some insight on the difference between kmer genome size estimation (776.06 Mb) and genome assembly size (863.59) with is close to 12%. 

 Regarding: "Based on the estimated genome size, the sequencing data provided approximately 60× coverage." If you divide 93.82 Gb by 0.776 Gb you obtain 120x and not 60. This should be checked.

 Looking at the Hi-C map, it is unclear how the W chromosome has been scaffolded. This should be explained. 

 The "primary only" kmer peak located at the homozygous position (60x) is not awaited. It usually indicates that your primary assembly contains duplicated haplotypic contigs. But the Busco scores do not show this duplication. This is really strange and should be checked. 

 The authors should use the same diagram type (meaning Y axis) for genomescope2 and multiplicity plot to ease comparison. 

 When you dot plot primary and alternate assemblies some of the chromosomes show configuration with very low probability of occurrence = over 10 rearrangements covering the complete chromosome. This is very unusual and should be validated by the Hi-C. The authors should provide Hi-C maps for both haplotypic chromosomes in such cases.

Are sufficient details of methods and materials provided to allow replication by others?

No

Is the rationale for creating the dataset(s) clearly described?

Yes

Are the datasets clearly presented in a useable and accessible format?

Yes

Are the protocols appropriate and is the work technically sound?

Yes

Reviewer Expertise:

genome assembly and annotation

I confirm that I have read this submission and believe that I have an appropriate level of expertise to confirm that it is of an acceptable scientific standard, however I have significant reservations, as outlined above.

# Reviewer report: Reviewer response for version 1

**DOI:** 10.21956/wellcomeopenres.29033.r157322

**Published:** 2026-06-15

**Authors:** Axel Künstner

**Affiliations:** 1 University of Lübeck, Lübeck, Germany

This is a standard Darwin Tree of Life genome note for *Pammene regiana*, following the well-established DToL template. The assembly metrics are strong: contig N50 of 9.25 Mb, scaffold N50 of 30.01 Mb, 99.44% of the sequence assigned to chromosomes, and BUSCO completeness of 98.6% (S:97.8%, D:0.7%). The EBP grade of 6.C.Q63 meets the reference standard.

 The assembly resolves 27 autosomes plus the W and Z. Counted conventionally (autosomes plus the Z), this is n = 28 - the modal number documented for Olethreutinae and specifically for the tribe Grapholitini, to which *P. regiana* belongs (Šíchová et al. 2013, PLoS ONE 8:e64520; cf. *Cydia pomonella*, n = 28, WZ/ZZ). The 29 pseudomolecules are this n = 28 complement plus the female-limited W. I would suggest the authors add a sentence placing the chromosome count in this clade-specific context: it is fully consistent with expectation for Olethreutinae and is not a reduction relative to the relevant baseline (n = 28 in this subfamily, versus n = 30 in Tortricinae and n = 31 ancestrally in Lepidoptera).This genome data note reports a chromosome-level reference assembly for Pammene regiana (Regal Piercer; Lepidoptera: Tortricidae: Olethreutinae), produced as part of the Darwin Tree of Life (DToL) project. The assembly was generated from a wild-caught female specimen from Wytham Woods, UK, using PacBio HiFi long reads for contig assembly (Hifiasm), Illumina Hi-C for scaffolding (YaHS), and supplementary RNA-seq data. The article follows the standard DToL data note format and contributes a high-quality reference genome for a species of interest as a representative of the speciose family Tortricidae and a herbivore associated with Acer.

 Summary of findings

 The primary assembly spans 863.59 Mb in 91 scaffolds, with 99.44% of sequence assigned to 29 chromosomal pseudomolecules representing 27 autosomes and the W and Z sex chromosomes. Key assembly metrics include a contig N50 of 9.25 Mb, scaffold N50 of 30.01 Mb, BUSCO completeness of 98.6% (single-copy 97.8%, duplicated 0.7%) against the lepidoptera_odb10 set, and an EBP summary grade of 6.C.Q63, exceeding the recommended 6.C.Q40 reference standard. The mitochondrial genome (16.5 kb) was also assembled. All raw data and assemblies are deposited under BioProject PRJEB71190.

 Major comments

 1. Chromosome count and phylogenetic context. The assembly resolves 29 pseudomolecules, comprising 27 autosomes, the Z, and the female-limited W. Counted in the conventional haploid notation (autosomes + Z), this corresponds to n = 28. The manuscript does not situate this number within the karyotypic context of the relevant clade, and`lepidopteran karyotype respectively, not of this subfamily. The chromosome complement of P. regiana is therefore fully consistent with expectation for its clade and does not represent a reduction. I recommend the authors add one or two sentences placing the observed n = 28 in this subfamily-level context, explicitly distinguishing it from the Tortricinae baseline that appears to motivate the reviewer's comment.

 Minor comments

 1. The k-mer completeness of the primary assembly alone (89.44%) falls below the EBP threshold of ≥95%, though the combined primary + alternate value of 98.15% is satisfactory. It would be helpful to add a brief sentence acknowledging this and noting that the combined assemblies meet the benchmark, as readers unfamiliar with haplotype-resolved assembly conventions may otherwise interpret the primary figure as a quality concern.

 2. Table 3 reports GC content rounded to 0.5% intervals for all but a few chromosomes. It is unclear whether this reflects the true precision of the estimates or a reporting convention. If the latter, a note to this effect would aid readers wishing to use these values.

 3. Manual curation involved 48 breaks and 58 joins, increasing scaffold count by 24.3% and assembly length by 0.8%. These figures are reported without further commentary. A brief statement on what types of misassemblies or joins were identified (e.g. chimeric contigs, proximity ligation artefacts) would add context, even if brief.

 Conclusion

 This is a technically sound and well-documented genome data note. The assembly quality is high by current standards and meets or exceeds EBP reference benchmarks. The primary concern — the interpretation of the chromosome count — is one of contextualisation rather than error, and can be addressed straightforwardly in a revised author response or in a subsequent version. I recommend approval subject to the addition of clade-appropriate karyotypic context.

Are sufficient details of methods and materials provided to allow replication by others?

Yes

Is the rationale for creating the dataset(s) clearly described?

Yes

Are the datasets clearly presented in a useable and accessible format?

Yes

Are the protocols appropriate and is the work technically sound?

Yes

Reviewer Expertise:

Genetics, Microbiome, Cancer

I confirm that I have read this submission and believe that I have an appropriate level of expertise to confirm that it is of an acceptable scientific standard.

# Reviewer report: Reviewer response for version 1

**DOI:** 10.21956/wellcomeopenres.29033.r153964

**Published:** 2026-05-05

**Authors:** Jurate De Prins

**Affiliations:** 1 National Collections and Marine Infrastructure, Commonwealth Scientific and Industrial Research Organisation (Ringgold ID: 2221), Canberra, Australian Capital Territory, Australia

It is very appreciated that the whole genome sequence is presented for this species. The authors found 29 chromosomal pseudomolecules, including W and Z chromosomes. This is one pair less than the usual set of chromosomes for microlepidoptera, which contains 30 pairs of chromosomes. Maybe the molecular data could provide a short explanation for this reduction?

 My second comment is actually related to all the articles of the Tree of Life Microlepidoptera section. It is very important to have 100% correct species ID of the specimen that was used for the whole mitogenome sequencing. A small photo of an adult in a rest position is certainly not enough. In this particular case, the ID was verified against DNA barcode database. It is *Pammene regiana, *otherwise I would doubt that it could also be *Pammene populana *(please see https://projects.biodiversity.be/lepidoptera/species/4349/)

 In the UK, and also in Flanders, every year about 20  species of Microlepidoptera are recorded that come to Europe and the UK with the plant and food trade from all over the world. Many of these species are not DNA barcoded, or some of them are misidentified or have the same DNA barcodes. Therefore, three independent character sets are used for correct species ID of the specimen, which is processed for whole mitogenome sequencing: 1) external characters; 2) male and/or female genitalia characters; 3) molecular characters. All three character sets should lead to the same species ID. Otherwise, the danger exists that an error or misidentification can occur in the ID of the whole mitogenomic set. Here, in this article is not the case, but I would advise including micromorphology traits for Microlepidoptera in the protocols. This is the most reliable method to recognise the exotic species arriving via human and trade travels, and also a double check for native European species of Microlepidoptera.

Are sufficient details of methods and materials provided to allow replication by others?

Yes

Is the rationale for creating the dataset(s) clearly described?

Yes

Are the datasets clearly presented in a useable and accessible format?

Yes

Are the protocols appropriate and is the work technically sound?

Yes

Reviewer Expertise:

Taxonomy, Microlepidoptera

I confirm that I have read this submission and believe that I have an appropriate level of expertise to confirm that it is of an acceptable scientific standard.
